# Training Intervention and Program of Support for Fostering the Adoption of Family-Centered Telehealth in Pediatric Rehabilitation: Protocol for a Multimethod, Prospective, Hybrid Type 3 Implementation-Effectiveness Study

**DOI:** 10.2196/40218

**Published:** 2022-10-28

**Authors:** Karen Hurtubise, Isabelle Gaboury, Jade Berbari, Marie-Claude Battista, Tibor Schuster, Michelle Phoenix, Peter Rosenbaum, Olaf Kraus De Camargo, Stacey Lovo, Lesley Pritchard-Wiart, Jill G Zwicker, Audrée Jeanne Beaudoin, Mélanie Morin, Thomas Poder, Marie-Pierre Gagnon, Geneviève Roch, Danielle Levac, Michel Tousignant, Heather Colquhoun, Kimberly Miller, Jennifer Churchill, Paula Robeson, Andréa Ruegg, Martine Nault, Chantal Camden

**Affiliations:** 1 School of Rehabilitation Sciences Faculty of Health Sciences McMaster Univeristy Hamilton, ON Canada; 2 Department of Family Medicine and Emergency Medicine Faculty of Medicine and Health Sciences Université de Sherbrooke Longueuil, QC Canada; 3 CanChild Centre for Childhood Disability Research School of Rehabilitation Sciences McMaster University Hamilton, ON Canada; 4 Centre de Recherche du Centre Hospitalier Universitaire de Sherbrooke Sherbrooke, QC Canada; 5 Department of Medicine Faculty of Medicine and Health Sciences Université de Sherbrooke Sherbrooke, QC Canada; 6 Department of Family Medicine McGill Univeristy Montreal, QC Canada; 7 Department of Pediatrics McMaster University Hamilton, ON Canada; 8 School of Rehabilitation Sciences University of Saskatchewan Saskatoon, SK Canada; 9 Department of Physical Therapy University of Alberta Edmonton, AB Canada; 10 Department of Occupational Sciences and Occupational Therapy University of British Columbia Vancouver, BC Canada; 11 BC Children's Hospital Research Institute Vancouver, BC Canada; 12 Institut Universitaire de Première Ligne en Santé et Services Sociaux Centre Intégré Universitaire de Santé et de Services Sociaux de l'Estrie - Centre Hospitalier Universitaire de Sherbrooke Sherbrooke, QC Canada; 13 École de Réadaptation Faculté de Médecine et Sciences de la Santé Univeristé de Sherbrooke Sherbrooke, QC Canada; 14 School of Public Health Université de Montréal Montréal, QC Canada; 15 Centre de Recherche de l'Institut Universitaire en Santé Mentale de Montréal, Centre Intégré Universitaire de Santé et de Services Sociaux de l'Est de l'Île de Montréal Montréal, QC Canada; 16 Population Health and Optimal Health Practices Research Unit, Centre Hospitalier de Québec-Univeristé Laval Research Centre Québec, QC Canada; 17 Faculty of Nursing Univeristé Laval Québec, QC Canada; 18 Faculty of Medicine Univeristé de Montréal Montréal, QC Canada; 19 Occupational Science and Occupational Therapy Department University of Toronto Toronto, ON Canada; 20 Department of Physical Therapy Univeristy of British Columbia Vancouver, BC Canada; 21 Empowered Kids Ontario East York, ON Canada; 22 Children's Healthcare Canada Ottawa, ON Canada; 23 Training Intervention and Program of Support Study Team Université de Sherbrooke Sherbrooke, QC Canada

**Keywords:** telehealth, pediatric rehabilitation, training, therapists, pediatrics, physical therapy, health care, family-centered care

## Abstract

**Background:**

Children with disability face long wait times for rehabilitation services. Before the COVID-19 pandemic, telehealth adoption was low across pediatric rehabilitation. Owing to the COVID-19 pandemic restrictions, pediatric therapists were asked to rapidly shift to telehealth, often with minimal training. To facilitate the behavior changes necessary for telehealth adoption, provision of appropriate evidence-based training and support is required. However, evidence to support the effective implementation of such training is lacking. The successful real-world implementation of a training intervention and program of support (TIPS) targeting pediatric therapists to enhance the adoption of family-centered telerehabilitation (FCT) requires the evaluation of both implementation and effectiveness.

**Objective:**

This study aimed to evaluate TIPS implementation in different pediatric rehabilitation settings and assess TIPS effectiveness, as it relates to therapists’ adoption, service wait times, families’ perception of service quality, and costs.

**Methods:**

This 4-year, pan-Canadian study involves managers, pediatric occupational therapists, physiotherapists, speech-language pathologists, and families from 20 sites in 8 provincial jurisdictions. It will use a multimethod, prospective, hybrid type 3 implementation-effectiveness design. An interrupted time series will assess TIPS implementation. TIPS will comprise a 1-month training intervention with self-paced learning modules and a webinar, followed by an 11-month support program, including monthly site meetings and access to a virtual community of practice. Longitudinal mixed modeling will be used to analyze indicators of therapists’ adoption of and fidelity to FCT collected at 10 time points. To identify barriers and facilitators to adoption and fidelity, qualitative data will be collected during implementation and analyzed using a deductive-inductive thematic approach. To evaluate effectiveness, a quasi-experimental pretest-posttest design will use questionnaires to evaluate TIPS effectiveness at service, therapist, and family levels. Generalized linear mixed effects models will be used in data analysis. Manager, therapist, and family interviews will be conducted after implementation and analyzed using reflective thematic analysis. Finally, cost data will be gathered to calculate public system and societal costs.

**Results:**

Ethics approval has been obtained from 2 jurisdictions (February 2022 and July 2022); approval is pending in the others. In total, 20 sites have been recruited, and data collection is anticipated to start in September 2022 and is projected to be completed by September 2024. Data analysis will occur concurrently with data collection, with results disseminated throughout the study period.

**Conclusions:**

This study will generate knowledge about the effectiveness of TIPS targeting pediatric therapists to enhance FCT adoption in pediatric rehabilitation settings, identify facilitators for and barriers to adoption, and document the impact of telehealth adoption on therapists, services, and families. The study knowledge gained will refine the training intervention, enhance intervention uptake, and support the integration of telehealth as a consistent pediatric rehabilitation service option for families of children with disabilities.

**Trial Registration:**

ClinicalTrials.gov NCT05312827; https://clinicaltrials.gov/ct2/show/NCT05312827

**International Registered Report Identifier (IRRID):**

PRR1-10.2196/40218

## Introduction

### Background

Timely access to family-centered services for children with disabilities and their families is crucial for supporting their development and well-being [1-3]. Currently, many children face long wait times (ie, up to 2 years) as well as organizational, geographic, or cultural barriers to services [4-6]. Lack of service access can lead to negative developmental, health, and social consequences for children and their families [7-10]. The COVID-19 pandemic further exacerbated these issues, as rehabilitation support for children was significantly reduced [11], increasing parental mental health burden (eg, stress and depression) [7-10]. To minimize the negative impacts of these service disruptions, therapists shifted to telehealth service delivery [7,9,12,13].

Telehealth is defined as any asynchronous or real-time clinical intervention provided remotely by clinicians (in this case, therapists) to patients or caregivers [14-16]. Telehealth is an important alternative for families living in underserved or remote areas [14,17-21]. However, some families in well-served urban locations also prefer the convenience of telehealth over in-person visits for reasons such as decreased travel time and schedule flexibility [15,16]. Before the COVID-19 pandemic, a systematic review of randomized controlled trials of pediatric rehabilitation delivered via telehealth supported the efficacy of rehabilitation provided via telehealth for diverse populations and a wide range of effects, including improved service access, child outcomes (eg, behavior), and family satisfaction [22]. Telehealth interventions have yielded promising results [23,24], and the acceptability [17,25-28] of telehealth has been previously established, further supporting its integration into comprehensive family-centered services [17,27,29,30].

Despite its established efficacy, the adoption of telehealth is low across rehabilitation, including in pediatric patients. An international survey conducted in August 2019 involving 1133 pediatric therapists from 76 countries reported that 3.9% of the pediatric therapists were using telehealth. However, in a follow-up survey completed in May 2020 (ie, during the public health restrictions imposed by the COVID-19 pandemic) with a subsample, 70.1% of the pediatric therapists had adopted telehealth. Many reported doing so without prior experience and lacked confidence, knowledge, and training in effective intervention strategies [12]. When asked about the support required to implement telehealth, training was by far the most frequently cited, and elements of training considered important included communication skills with families over the telephone and internet, safe and effective use of platforms, reliable assessment tools and processes, and intervention strategies for children of various ages and health conditions [12]. Although therapists’ knowledge, skills, and attitudes toward telehealth can improve with time and experience [31], training and support are required for behavioral changes to occur [32-34]. Unfortunately, there is a paucity of evidence on how personal and contextual factors may influence telehealth training and support [35]. Targeting therapists’ knowledge, skills, and attitudes associated with their intention to adopt telehealth and their role within family-centered services appears vital to the effective implementation of telehealth [35-43].

Family-centered telerehabilitation (FCT) is defined as pediatric rehabilitation that uses family-centered care practices while working with families remotely. Family-centered care is recognized as the best practice approach in pediatric rehabilitation [44]. Described as a partnership approach, family-centered care is based on the belief that the child’s well-being and care needs are best supported within the family context through effective family-provider collaborations [45]. A central family-centered care tenet is the assumption that the processes of care delivery are as important to child and family outcomes as the specific characteristics of the clinical intervention delivered [45]. Family-centered care is characterized by practices that promote clinical flexibility; respect and dignity for families’ perspectives, knowledge, strengths, and characteristics; effective information sharing (general and specific), partnership, and collaboration among parties to support decision-making; and coordinated and comprehensive care delivery [30]. Furthermore, family-centered care occurs in therapeutic environments that optimize the development of family-provider partnerships [46-49], in which parents are active participants in collaborative goal-setting therapy [50,51], planning, implementation, and evaluation [44,46,52,53] and where activities are integrated within daily routines and contexts such as home and community [54].

Telehealth offers additional opportunities to enhance family-centered care practices [30,55] as it provides convenient and flexible ways to partner with families, respecting individual family composition, characteristics, and constraints (eg, geographical, temporal, and financial) [21]. Furthermore, it allows real-time knowledge acquisition and information sharing about the child within their contexts and supports family decision-making and parents’ psychosocial well-being such as decreased anxiety, stress, and depression [21]. Finally, telehealth has been recognized as an important addition to comprehensive care coordination and service delivery [56].

As a result of the pandemic, considerable momentum exists to support the uptake of FCT and foster its ongoing sustainable use within accessible and supportive services for the families of children with disabilities. Pediatric rehabilitation therapists, service managers, professional associations, policy makers, and patients are calling for resistance to *returning to normal* and instead are requesting help to sustain telehealth as part of the FCT continuum of care [28,29,56,57]. For this shift to occur, therapists require tools, training, and support. The proposed study aims to evaluate the implementation of a training intervention and program of support (TIPS) to enhance the adoption of FCT in pediatric rehabilitation centers across Canada and to assess its impact on wait times, families’ perception of service quality, and costs.

### Intervention

**Figure 1:**
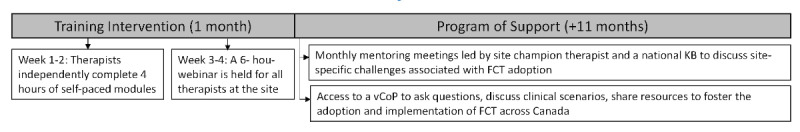
Training intervention and program of support description. FCT: family-centered telerehabilitation; KB: knowledge broker; vCoP: virtual community of practice.

TIPS is an evidence-informed, multifaceted intervention, informed by empirical evidence in the field of pediatric rehabilitation and effective implementation strategies [16,58-62]. TIPS consists of the following components: (1) a 10-hour intensive training program offered to participating therapists at each site over a 1-month period, which includes 4 hours of self-paced learning modules and a 6-hour mandatory webinar and (2) an 11-month program of support composed of monthly mentoring meetings at each site led by the local therapist champion and a national, virtual community of practice. The virtual community of practice will be offered simultaneously to all participating therapists across Canada and facilitated by 3 national knowledge brokers—an occupational therapist, a physiotherapist, and a speech and language pathologist—experienced in FCT in pediatric rehabilitation. Figure 1 illustrates components and time frame of TIPS.

The TIPS self-paced learning modules are informed by contemporary family-centered care frameworks and the family-oriented service continuum [30]. More specifically, they will address FCT core components and provide practice examples. Modules will address the following topics, as they apply to telehealth: (1) overview of family-centered care premises and principles (eg, information provision; respectful, supportive, and comprehensive care; and enabling partnerships) [63,64], (2) parent-professional collaborative partnerships (eg, goal cocreation, parent and child engagement, and role negotiation) and helpful FCT instruments and strategies [65], (3) coaching in the FCT context (eg, various approaches and strategies) [52,66], and (4) factors influencing service delivery model choice (ie, face-to-face or telehealth) [12]. As per best practice [16] and using education course creation software, several members of the study team will codevelop multimedia content (eg, videos and presentations) for the asynchronous training in consultation with pediatric clinicians and other experts (eg, parent partners, national organizational partners, inclusion and diversity experts, and knowledge keepers). We will upload the curriculum to a password-protected web-based platform for which a unique username and password will be required. Knowledge acquisition, based on specified learning objectives and key messages targeted in each training module, will be assessed through short quizzes. Completion of the asynchronous modular training and knowledge assessment will be recommended before undertaking the synchronous webinar.

A 6-hour synchronous webinar component will also be delivered to participants by members of the research team and 3 knowledge brokers. The webinars will engage therapists in discussions using case studies and interactive activities (eg, role play, vignettes, and simulations) to build their critical thinking on how to implement these practices in their context and with the families they serve. The webinar content will be adapted for each site in consultation with the local leadership team (ie, a site manager, a therapist champion, and a parent or patient partner). This coadaptation phase will ensure that activities and practice examples are tailored to individual site contexts and processes (eg, engagement practices, site clinical goal-setting processes, and service coordination as per team procedures) and that webinars are learner-centered and clinically relevant. Therapists will be encouraged to consider various asynchronous and real-time technologies, including email, telephone, web-based platforms, and videoconferencing systems that best respond to families’ needs and preferences and are approved by their organizations. The research team will refrain from recommending specific technologies. There will be no prescribed frequency or duration for the FCT interventions; rather, therapist participants will work with families according to their goals and preferences, and site-specific organizational policies.

Finally, a program of support will be offered for the remaining year via monthly videoconference mentoring meetings and access to the virtual community of practice, which will be housed on the password-protected web-based platform. Monthly meetings will focus on sharing site-specific successes and challenges, proposing solutions and reporting results, as well as sharing practical evidence-informed resources. The evidence-informed virtual community of practice, facilitated by the 3 national knowledge brokers, will be used to canvas for solutions to address challenges at a national level; share successes; discuss specific cases for guidance, feedback, and input; and share useful tips, tricks, and resources [43,59,67-71].

### Research Question and Study Objectives

Our hybrid implementation-effectiveness study examining the implementation of TIPS aims to answer the following research question: *Can TIPS enhance the adoption of FCT interventions by therapists working in different contexts?*

Specific objectives of the study include the following:

1. Implementation evaluation primary objectives: to assess therapists’ intention to adopt FCT practices and evaluate therapists’ fidelity to FCT practices
2. Implementation evaluation secondary objectives: to document the contextual variations required to coadapt TIPS to meet each site’s needs and identify factors influencing FCT adoption and fidelity

For the implementation evaluation, we hypothesize that, in the short term (ie, 1 month after TIPS), therapists’ intention to adopt FCT will increase minimally and their fidelity to FCT practices will improve minimally. After the implementation of TIPS (ie, >1 month), we expect that FCT adoption will increase and the fidelity of FCT practices will improve modestly. We also expect that engagement will fluctuate over time, across sites and therapists and will depend on therapist, client, organizational, and system factors.

1. Effectiveness evaluation: to compare service wait times, families’ perceptions of service quality, and changes in service delivery before and after the implementation of TIPS and explore the costs (and cost savings) related to increased use of FCT

For the effectiveness evaluation, we hypothesize that for sites with the largest effect change in intention to adopt FCT and the fidelity of FCT practices, (1) wait times will significantly decrease and (2) families’ perceptions of service quality will significantly improve after the implementation of TIPS. In relation to cost, we also expect families to experience cost savings after the implementation of TIPS and managers to report no additional costs incurred because of TIPS.

## Methods

### Study Design

The TIPS study is a 4-year, multimethod, hybrid type 3 implementation-effectiveness trial, registered with ClinicalTrials.gov (NCT05312827). Hybrid implementation-effectiveness trial designs are recommended when the traditional research pipeline of efficacy-effectiveness-implementation is too time-consuming and considered unethical, failing to adequately respond to the urgency of the expressed need [72]. TIPS is well suited to this type of hybrid implementation-effectiveness design because (1) there is momentum for its implementation within the health care system, (2) minimal risk is associated with the clinical intervention and the implementation strategy to support generalizability, (3) there is strong face validity and indirect evidence for the clinical intervention and implementation strategy to support generalizability, and (4) there is evidence of feasibility for the implementation strategy and support in the clinical and organizational context under study [72]. A prospective, hybrid type 3 design reflects a collaborative ethos because it allows end users to inform the refinement and improvement of clinical interventions and their implementation processes [72,73]. Implementation strategies will be adjusted during the intervention refinement process in consultation with parents of children with a disability, clinicians supporting these families, individuals with experience implementing digital health, including health services managers, as well as the pediatric rehabilitation implementation sciences literature. Potential additional user-identified strategies will be integrated as part of the consultation process and according to the collaborative approach adopted. These strategies may allow previously unrecognized FCT implementation barriers to be acknowledged and addressed.

### Study Settings

Participating sites are publicly funded organizations providing outpatient pediatric rehabilitation or child development services to children aged 0 to 12 years with, or at risk of, disability. *Disability* is used inclusively to recognize all medical diagnoses associated with limitations in functioning, such as cerebral palsy and autism spectrum disorder. The term *at risk* includes children presenting with delayed development who may not yet have a diagnosis but who experience functional limitations and qualify for rehabilitation services. The upper age limit of 12 years was chosen, as best practices regarding transition of care suggest that different relationships should be fostered with adolescents aged >12 years [74].

The 20 participating sites were selected to be representative based on various characteristics (eg, population, size, services provided, catchment area, and geography) posited to influence outcomes, the effects of which will be explored. These sites are clustered into 6 regions (one of which includes 3 provinces with a single participating site). To limit the risk of contamination, and as per the interrupted time series design, TIPS will be implemented in all sites in the same region during the same month and sequentially introduced across all regions, 2 months apart. Training will be conducted on a site-by-site basis to create team cohesion. The 2-month implementation interval between regions provides flexibility for organizing implementation and data collection activities.

### Participants

Participants will be recruited from study sites according to the following eligibility criteria:

1. Managers (n=20; one per site): managers, or their delegates, responsible for rehabilitation services at the site and members of the local leadership team, will participate in the coadaptation of TIPS to their site. Managers may contribute to their site’s monthly mentoring meetings; aid in the recruitment of therapists, parents, a therapist champion, and a parent-partner for their site; and complete site- or service-specific data collection instruments before and after the implementation of TIPS.
2. Therapists (n=600 with 50% anticipated response; n=300): physiotherapists, occupational therapists, or speech-language pathologists providing outpatient pediatric rehabilitation services to children aged 0 to 12 years at each site are recruited via the managers and are interested in using FCT. Therapists will participate in the TIPS program, complete data collection instruments as prescribed, and aid in parent recruitment.
3. Parents (n=20 per therapist with an anticipated response rate of 33%; n=2000 families per assessment time point): 1-time data collection will be undertaken with 2 samples (preimplementation and postimplementation samples) of parents or caregivers who received services (either in-person, virtually, or both) from at least one participating therapist in the previous 3 months.
4. Therapist champion (n=20; one per site): a therapist selected based on their telehealth experience and on peer recognition within their organization. Therapist champions are members of the local leadership team participating in the coadaptation of TIPS to their site and will oversee the monthly mentoring meetings and agree to report on the implementation process after the implementation of TIPS.
5. Parent or patient partner (n=20; one per site): parent or patient partners will primarily be recruited from family, parent, or patient advisory committees at the participating sites or, in the absence of such initiatives, from regional, provincial, or national patient engagement programs. As members of the local leadership team, parent or patient partners will participate in the coadaptation of TIPS to their site and could be called upon to contribute to their site’s monthly mentoring meetings.

Recruitment procedures will be flexible and will be adapted to the preferences, policies, and procedures at each site. The recruitment of participants may be undertaken by email and sent directly to the potential participant by the research team (eg, therapists) or by the manager or therapist on behalf of the research team (eg, parents).

### Sample Size

#### Number of Sites

A total of 20 sites across 8 Canadian provinces (grouped into 6 regions for the intervention rollout) are included in the study. Whenever possible, at least 3 sites per province were included to ensure sample diversity, enable the exploration of the provincial health systems’ effects on the outcomes, and estimate site-related variations in outcomes. A total of 5 regions will include sites in the same province, whereas 1 region will consist of sites from 3 different provinces where only 1 rehabilitation program is available.

#### Number of Therapists

At the therapist level, implementation outcomes will be assessed 3 times during each period (before, during, and after the implementation of TIPS) for a total of 10 data collection time points. Assuming an autocorrelation of repeated measures of *r*<0.3, data collected from 300 therapists will provide >80% power to detect moderate effect sizes (Cohen *d*≥0.5), using a first-order autoregressive segmented regression model [75] and a global type I error level of 5%, accounting for multiplicity of outcome assessments (Šidák correction) [76].

#### Number of Families

With a minimum expected sample size of 20 families per therapist being assessed before and after the implementation of TIPS, statistical power will be >90% to detect even small effect sizes (0.1<Cohen *d*<0.3) for the effectiveness outcomes (ie, change in wait times and change in families’ perceptions of service quality).

### Conceptual Framework

**Figure 2:**
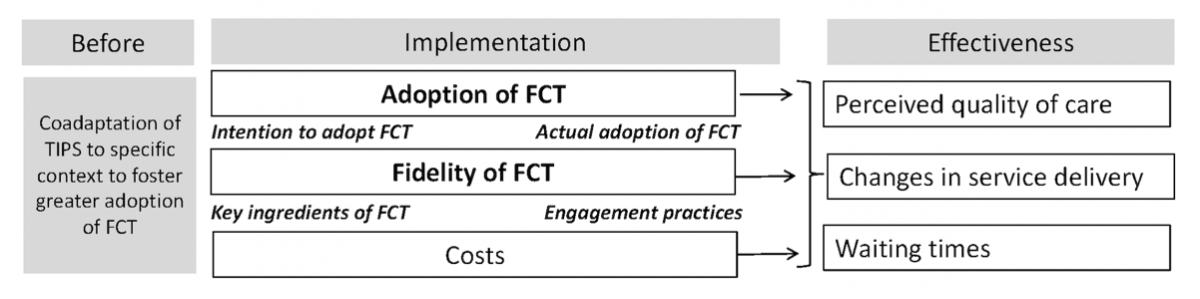
Conceptual framework and key concepts as per implementation-effectiveness design. FCT: family-centered telerehabilitation; TIPS: training intervention and program of support.

The structure of this study (Figure 2) builds on implementation science frameworks that aim to accelerate the translational research pipeline [72], bridging the current knowledge-to-practice gap. Specifically, the Consolidated Framework for Implementation Research [77] will guide the identification of factors influencing the adoption of FCT and will help engage leaders in participating sites in adapting the TIPS to their own contextual drivers, while maintaining the FCT key ingredients. A type 3 hybrid design will be used, primarily focusing on implementation indicators (bold text in Figure 2), while also collecting some effectiveness outcomes, with comparative assessments occurring at the therapist, service, or family level. This design is recommended when there is (1) momentum for implementation within the health care system, (2) strong face validity and indirect evidence for the clinical intervention and implementation strategy to support generalizability, (3) minimal risk associated with the clinical intervention and the implementation strategy, and (4) evidence of feasibility for the implementation strategy and support in the clinical and organizational context under study [72].

Data collection procedures are presented in Figure 2, and Table 1 presents an overview of the tools used and the participant groups involved. Details are provided in the next sections.

**Table 1:** Data collection measures, targeted participants, and time points.

Objective and measures				Participants		Time points																			
						Before TIPS^a^						During TIPS									After TIPS				
						T–3^b^		T–2^c^		T–1^d^		T0^e^		T1^f^		T2^g^		T3^h^		T4^i^			T5^j^		T6^k^
**Implementation evaluation**																									
	Intention to adopt: ACCEPT-VFCC^l^		Therapists		✓		✓		✓		✓		✓		✓		✓		✓			✓		✓	
	**Fidelity to FCT^m^ practices**																								
		FCT fidelity checklist	Therapists		✓		✓		✓		✓		✓		✓		✓		✓			✓		✓	
		PRIME-SP^n^	Therapists		✓		✓		✓		✓		✓		✓		✓		✓			✓		✓	
	**Documentation of TIPS coadaptation**																								
		Organizational readiness for eHealth questionnaire	Managers		✓																				
		Group discussion recordings	Leadership team				✓																		
	**Influencing factors**																								
		Monthly therapists’ meetings recordings	Therapists										✓		✓		✓		✓						
		Virtual community of practice discussion threads	Therapists										✓		✓		✓		✓						
**Effectiveness evaluation**																									
	Service wait times: time from service eligibility to first scheduled appointment		Managers		✓																	✓			
	Perceptions of service quality: MPOC-20^o^		Parents		✓																	✓			
	Changes in service delivery: semistructured interviews		Managers, therapist champions, therapists, and parents																			✓			
	**Costs**																								
		Organizational costs	Managers																			✓			
		Family costs	Parents		✓																	✓			
		Implementation cost (cost journal)	Research team				✓		✓		✓		✓		✓		✓		✓						

^a^TIPS: training intervention and program of support.

^b^T–3: 3 months before implementation.

^c^T–2: 2 months before implementation.

^d^T–1: 1 month before implementation.

^e^T0: implementation initiation.

^f^T1: 1 month after implementation + end of training intervention.

^g^T2: 4 months after implementation.

^h^T3: 8 months after implementation.

^i^T4: 12 months after implementation + end of support program.

^j^T5: 15 months after implementation.

^k^T6: 18 months after implementation+6 months after end of implementation.

^l^ACCEPT-VFCC: Assessment of Competencies and Contributors to Enhance Practice Transition to Virtual Family Centered Care Survey.

^m^FCT: family-centered telehealth.

^n^PRIME-SP: Pediatric Rehabilitation Intervention Measure of Engagement-Service Provider version.

^o^MPOC-20: Measure of Processes of Care-20.

### Implementation Evaluation

#### Overview

The implementation evaluation will primarily seek *to assess therapists’ intention to, and adoption of FCT, and their fidelity to FCT practices* (ie, objective 1). An interrupted time series was selected to assess primary implementation outcomes as recommended for research in real-world settings [78,79]. Unlike other design types that rely on randomization (eg, stepped wedge), this design allows for site leadership teams to be included in the discussion about the timing of the initiation of the intervention [80]. An interrupted time series consists of observing the same dependent variables over time with a break in the series of observations corresponding to the introduction of an intervention. If the intervention is effective, a change in the series’ pre- and postintervention averages will be observed [79]. TIPS will be implemented in all sites in a specified jurisdiction during the same month and sequentially introduced across all jurisdictions, 2 months apart. Implementation data will be collected at least 3 times during each study period [81]: before (T–3 to T–1), during (T0 to T3), and after the implementation of TIPS (T4 to T6). The additional data collection time point at 1 month following the TIPS implementation will allow documentation of the short-term impact of the *training intervention* portion of TIPS (ie, the self-paced modules and webinar). Statistical analysis models will account for the inequivalent time intervals across study periods [75].

#### Data Collection

The *therapist’s implementation questionnaire*, completed electronically by therapists at multiple time points (ie, T–3 to T6), will include questionnaires addressing the *primary objectives* and will comprise the measures discussed next.

To compare changes in therapists’ intention to, and adoption of, FCT (ie, objective 1), the *Assessment of Competencies and Contributors to Enhance Practice Transition to Virtual Family Centered Care survey* will be used. This measure, based on the validated Theoretical Domain Framework Questionnaire template [81-83], examines 8 constructs across 41 items and considers factors, including knowledge and skills, social or professional role identity, beliefs in capacity-building attitudes, therapists’ intention to adopt a virtual practice based on FCT, and environmental, patient-targeted, and other factors perceived to affect FCT implementation. Therapist participants will be asked to rate their responses to 36 specific statements associated with each theoretical domain framework domain using a 7-point Likert scale (1=strongly disagree to 7=strongly agree). A total of 4 open-ended or multiple-choice questions related to identification of facilitators and barriers and training preferences round out the instrument. Furthermore, the number of FCT sessions conducted in the proceeding months will document the actual adoption of FCT by therapists. Upon the initial completion (ie, at T–3), participants will be asked to respond to questions associated with sociodemographic characteristics (eg, professional education and work experience) and their previous telehealth experience, including prior training and use. This section will not be repeated at subsequent data collection time points (ie, T–2 to T6).

To monitor therapist fidelity to FCT practices (ie, objective 1b), the *FCT fidelity self-perceived checklist* and *Pediatric Rehabilitation Intervention Measure of Engagement-Service Provider version (PRIME-SP)* will be used. The *FCT fidelity self-perceived checklist*, a 4-item instrument, developed and pilot tested in a previous study [84], will measure therapists’ perceptions of the perceived quality of 3 interventional behaviors associated with the FCT clinical intervention (ie, goal focused, active parent partnerships, and evidence of supportive and trusting parent-professional relationships) based on their last telehealth session. Each behavior, comprising 4 criteria, is scored on a 5-point Likert scale (0=behavior not implemented when it should have been to 4=all the identified behaviors were implemented when appropriate and within context). The fourth item assesses the representability of the chosen session to a typical parent-therapist interaction. The *PRIME-SP* will be used to measure therapists’ views on how engagement practices were implemented with clients during their last telehealth session and the clients’ responses [85]. It consists of an overall rating, as well as separate ratings of affective, cognitive, and behavioral aspects of a client’s engagement state, based on a 4-point descriptive scale (disengaged to extremely engaged). Face, content, and construct validity of the PRIME-SP have been established [85].

#### Data Analysis

To evaluate implementation (ie, objective 1), longitudinal mixed modeling accounting for and considering potential methodological issues associated with an interrupted time series analysis (eg, autocorrelation and time-varying confounders) will be used to analyze implementation indicators (ie*,* Assessment of Competencies and Contributors to Enhance Practice Transition to Virtual Family Centered Care survey, therapists’ self-reported FCT frequency, FCT fidelity self-perceived checklist, and PRIME-SP). Changes will be documented in the short term (ie, 1 month after TIPS introduction) and in the long term (ie, at the end of TIPS, 12 months after its introduction). Models will be covariate-adjusted to reduce potential confounding bias, including the therapists’ characteristics (eg, gender and years of experience) and site characteristics (eg, service provided, geography, and general patient characteristics), to estimate associations of key explanatory variables alongside TIPS. Secondary analyses will explore the heterogeneity in changes of outcome measures across genders, sites, therapists, and health jurisdiction levels.

*Secondary implementation objectives* will be evaluated using a multimethod approach.

#### Data Collection and Analysis

The local leadership team, involved in *documenting the coadaptation of TIPS* (ie, objective 2), will be asked to complete a sociodemographic questionnaire to record their characteristics such as years of experience, level of expertise, and previous experience with telehealth services and technologies. An initial draft logic model will be developed by the research team based on the best evidence related to knowledge translation strategies to best address the FCT needs identified by therapists in a national survey and in recent publications. This draft will subsequently be presented to the local site team members for feedback. A *discussion group format* [86] will be used to gather the local leadership team members’ input, which will then be used to coadapt TIPS (ie, logic model, training curriculum, and materials) to site-specific needs. Throughout the coadaptation process, discussions will be audio recorded, meeting documents will be collected, and proposed adaptations and decisions made by local leadership team committee members, and their reasoning for these modifications will be recorded during the discussion group in real-time and in the TIPS logic model, training curriculum, and materials.

To *identify the factors influencing therapists’ intention to adopt and use FCT* (ie, objective 3), *monthly mentoring meeting, audio recordings* and *materials* (eg, meeting agendas and suggested resources), *virtual community of practice discussion thread content*, and *semistructured interview audio recordings* with participating managers, therapist champions, therapists, and families after the implementation of TIPS will be collected. Data will be analyzed thematically using a deductive-inductive approach guided by the Consolidated Framework for Implementation Research domains [77].

### Effectiveness Evaluation

#### Overview

To *evaluate TIPS effectiveness* (objective 4) and *costs* (objective 5), a mixed methods pre-post design has been chosen to measure the intervention effectiveness outcomes and costs using easily accessible service indicators and questionnaires administered to parents. To capture additional effects, semistructured interviews will also be conducted after the implementation of TIPS, with all participant groups. Instruments and processes are described in detail in further sections.

#### Data Collection

At the service level, the *site profile questionnaire*, completed by managers before and after the implementation, includes questions related to organizational readiness for eHealth [87] as well as clinically relevant *wait time indicators* (eg, the average service wait time for service) [88,89]. To estimate changes in wait times (before vs after), a confounder-adjusted analysis using generalized linear mixed effects models will use a log-link function to account for the typically right-skewed nature of time data. Estimated fixed (intervention) effects for the effectiveness outcomes will be reported with Šidák-corrected 95% CIs [76].

To evaluate changes in perceived service quality, the *family questionnaire* will be electronically distributed by managers or therapists to eligible families. It includes a sociodemographic questionnaire (eg, remoteness of location), and *Measurement of Processes of Care-20* (MPOC-20), a valid and reliable 20-item self-reported measure of parents’ perceptions of the extent to which rehabilitation services are family-centered [90]. It contains five scales: (1) enabling and partnership, (2) providing general information, (3) providing specific information about the child, (4) coordinated and comprehensive care for the child and family, and (5) respectful and supportive care; it is scored on a 7-point Likert response scale, which indicates the extent to which the service provider engaged in the behavior (1=not at all to 7=to a very great extent). Each scale yields its own score, and no total score is calculated. Data will be analyzed using generalized linear mixed effects models with nested random effects (families within therapists within sites) to control for the correlated nature of the data (ie, the possibility that families have responded once or twice to the MPOC-20) and to account for therapist and site cluster effects. Analyses will be conducted for each of the 5 MPOC-20 domains and controlled for the same confounding variables described in the analysis for objective 1, as well as for family-level variables (eg, sociocultural background, child’s age, and gender).

To explore all changes in service delivery (both negative and positive), participants will be invited to participate in audio-recorded *semistructured interviews* after the implementation of TIPS. The sample will include all managers, some therapists (all local site champions and a subsample of therapists showing high or low adoption in different sites), and parents with diverse sociocultural characteristics, levels of perception of quality of care, and experience with FCT. Interview data will be analyzed thematically using an inductive approach to better understand the breadth and depth of changes to pediatric rehabilitation service delivery according to various stakeholder perspectives. Integration of quantitative and qualitative data using the aforementioned explanatory approach [91] will allow us to uncover the anticipated and unanticipated effects of FCT on pediatric rehabilitation service delivery.

To *explore costs*, an economic evaluation following a health care perspective as recommended by the Canadian Agency for Drugs and Technologies in Health [92,93] will be used primarily. The research team will maintain a *costs journal* related to TIPS implementation (eg, knowledge brokers’ salary). Costs relating to therapist participation in TIPS (time × average salary) and those resulting from changes in the organizational setting (eg, telehealth equipment) will be documented in the managers’ *site profile questionnaire*. *Families’ costs and savings*, including impact on travel time, parking costs, missed work, and costs related to equipment or internet, will be included in the *family questionnaire*. The total costs related to the implementation of the TIPS will be calculated (ie, additional therapists and knowledge brokers’ time and salary), as well as costs per participating therapist and costs per site accounting for different organizational characteristics. Relative cost, an estimation of costs per session, cost per client seen by the therapist, and incremental ratios (ie, change in costs to use the TIPS divided by change in the primary implementation outcomes measures and secondary effectiveness measures) will also be computed. Finally, societal costs (ie, savings for families in decreased travel time, parking, missed work, and costs related to equipment or internet) will be explored for robustness analysis.

### Ethics Approval

The research ethics committees overseeing the 20 participating sites will approve this research project. At the time of manuscript submission, the Research Ethics Board of the *Centre intégré de santé et des services sociaux de l’Estrie—Centre hospitalier universitaire de Sherbrooke* approved this project (ID MP-31-2022-4546) along with the Health Research Ethics Board-Health Panel at the University of Alberta (ID Pro00119976). Ethics approvals have also been submitted to the Hamilton Integrated Research Ethics Board and the Human Ethics Board at the University of British Columbia. Finally, ethics submissions are in preparation for the Interdisciplinary Committee on Ethics in Human Research at the Memorial University of Newfoundland, the Izaak Walton Killam Research Ethics Board, the University of Manitoba Ethics Board, and the University of Saskatchewan Human Ethics Board. Informed consent will be obtained before data collection from the participating managers, therapists, and parents. Participants will be informed that the study data will not constitute an evaluation of their professional performance. Data collection will occur entirely on the web using secure data collection and management solutions.

## Results

Funding was provided by the Canadian Institutes for Health Research on July 22, 2021. All 20 sites were recruited for the funding application. Ethics approval for the first participating site (ID MP-31-2022-4546) was received in February 2022 and for the second site (ID Pro00119976) in July 2022; submissions are either in preparation or pending in the other jurisdictions. To prepare sites, manager meetings were conducted between October 2021 and November 2021 to review responsibilities (eg, identification of site leadership members) and discuss timing for study initiation. As a result, data collection is anticipated to start in September 2022 and conclude by September 2024. Data analysis will occur concurrently with data collection until late 2024. Study- and site-specific results will be available for dissemination from early- to mid-2025, with publications available throughout the same year.

## Discussion

### Overview

Telehealth is a feasible, acceptable, and cost-effective service delivery option for pediatric rehabilitation for children experiencing, or at risk for, disability, and has established effectiveness in improving service access, child outcomes, and family satisfaction with pediatric rehabilitation [12,15,16,19,28,29]. However, before the pandemic, adoption in pediatric rehabilitation was low [12,35]. Despite the recent rapid uptake and dramatic increase in the use of telehealth owing to the public health restrictions imposed by the pandemic, many pediatric therapists provided telehealth without appropriate training and support [12].

To fill this gap, this hybrid implementation-effectiveness study aims to (1) evaluate whether the implementation of TIPS will enhance the adoption of FCT interventions by therapists working in different contexts and the contextual factors that may influence their adoption, (2) assess TIPS effectiveness on wait times and families’ perceptions of service quality, and (3) explore costs from a health care perspective. Therapists’ intention to adopt FCT is expected to increase minimally in the short term (ie, 1 month after the implementation of TIPS), as is their fidelity to FCT practices. Modest increases in adoption and in fidelity in the longer term (ie, >1 month after TIPS), with fluctuating engagement over time dependent on therapist, family, organizational, and system factors are anticipated. For sites with the largest effect change in intention to adopt FCT and fidelity of FCT practices, it is hypothesized that wait times will significantly decrease, whereas families’ perceptions of service quality will significantly improve after TIPS implementation. Finally, families’ cost savings after TIPS are anticipated, with managers reporting no additional cost incurred because of TIPS.

Moreover, we hope that this study will generate the knowledge required on how to support therapists in implementing FCT practices within pediatric rehabilitation services. We will also identify the contextual factors that may influence therapists’ adoption of telehealth and affect telehealth effectiveness at therapist, service, and family levels. As a prospective study, this knowledge will be contextualized to support therapists working in varied settings, building local capacity, and ensuring pediatric therapists have the established skills needed to deliver FCT interventions effectively. Study- and site-specific findings will be disseminated to organizational partners via webinar presentations. All training materials will be made readily available across Canada and internationally to facilitate the development of telehealth knowledge and skills more broadly in the current and upcoming national and international pediatric rehabilitation workforce. Training materials, implementation strategies, and study findings may also assist pediatric rehabilitation organizations and their leaders in generating appropriate policies, ongoing training opportunities, and procedures to ensure sustained delivery of comprehensive, high-quality rehabilitation service models, which include telehealth as an option. Finally, the study findings may also be the catalyst for the development of a set of required competencies for physiotherapists, occupational therapists, and speech-language pathologists who use telehealth to deliver rehabilitation services. To ensure wide dissemination to a variety of interested audiences, the study results will be shared as publications, conference presentations, on social media, and via newsletters.

### Strengths and Limitations

The strengths of the TIPS study lie in its implementation in various real-world contexts and its use of a hybrid implementation-effectiveness approach. The multimethod design will allow for the inclusion of multiple implementation measures and an in-depth exploration of the contextual factors affecting the implementation and adoption of FCT. Finally, the multilevel (ie, service, organizational, and consumer) assessment of effectiveness will create a comprehensive overview of its impact. In addition to leveraging implementation science theory and evidence, the research processes are carefully designed to ensure the inclusion and integration of key stakeholder implementation knowledge at strategic moments (eg, before implementation and following the training) throughout the study, keeping the focus on the end users, to ensure implementation success. The participation of multiple and varied pediatric rehabilitation services allows for the examination of TIPS implementation and its impacts across various diverse real-world contexts that exist in Canada. It is hoped that the triangulation of a comprehensive range of both qualitative and quantitative data will provide useful insights into the wide range of factors affecting FCT implementation and adoption and the plethora of potential resulting effects. However, this study has some limitations. First, as recruitment is being facilitated through the site managers, the possibility of a considerable selection bias in participant recruitment (ie, therapists and parents) exists. The individuals approached may share similar characteristics, views, and perspectives on this service option, which could limit the variability in our sample. Second, this study relies on self-reported outcome measures, some of which have been developed specifically for this study, and for which psychometric properties are being assessed. Third, multiple data collection time points increase the risk for missing data. Some strategies have been planned to mitigate these constraints; those that persist will be acknowledged in the reporting of the results to assist in appropriate interpretation of the findings.

### Conclusions

The TIPS study will inform the contextual implementation of a training and support program to enhance the adoption of FCT. This study will assess the effectiveness of a training and support program in changing pediatric therapists’ FCT adoption, parents’ perceptions of service quality, service access wait times, and the cost associated with this service option. The study outcomes will increase pediatric rehabilitation service delivery options for families, improve access to services, and foster greater well-being for families of children with, or at risk of, disability.

## Appendix

Multimedia Appendix 1 Peer-review report by the Canadian Institutes of Health Research (CHIR)/Instituts de recherche en santé du Canada (IRSC) - Institut des services et des politiques de la santé / Institute of Health Services and Policy Research - Knowledge Translation Research/Recherche sur l'application des connaissances (Canada).

## Abbreviations

FCT: family-centered telerehabilitation
MPOC-20: Measure of Processes of Care-20
PRIME-SP: Pediatric Rehabilitation Intervention Measure of Engagement-Service Provider version
TIPS: training intervention and program of support

## Data Availability

The data sets generated or analyzed during this study will be available from the corresponding author upon reasonable request.
