# Supplementary material for: Training Intervention and Program of Support for Fostering the Adoption of Family-Centered Telehealth in Pediatric Rehabilitation: Protocol for a Multimethod, Prospective, Hybrid Type 3 Implementation-Effectiveness Study
Source: JMIR Res Protoc. 2022 Oct 28;11(10):e40218. doi: 10.2196/40218 (PMC9652740; doi:10.2196/40218)
Supplement: Multimedia Appendix 1 [file resprot_v11i10e40218_app1.pdf]

Canadian Institutes of Health Research/Instituts de recherche en santé du Canada

Notice of Recommendation/Avis de recommandation

Application Number/Numéro de la demande: 462626

Committee Code/Code du comité: KTR

Applicants/Candidats: Docteure Chantal Camden

With/Avec:

Professeure M. Battista	Madame A. Beaudoin	Ms. J. Churchill
Dr. H. Colquhoun	Professeure I. Gaboury	Docteure M. Gagnon
Ms. K. Hurtubise	Dr. O. Kraus De Camargo	Ms. D. Levac
Dr. S. Lovo	Dr. K. Miller	Docteure M. Morin
Madame M. Nault	Dr. M. Phoenix	Mrs. P. Robeson
Docteure G. Roch	Dr. P. Rosenbaum	Madame A. Ruegg
Dr. T. Schuster	Professeur M. Tousignant	Dr. L. Wiart
Dr. J. Zwicker		

Institution paid/ Établissement payé: Université de Sherbrooke

Title/Titre: Training Intervention and Program of Support (TIPS) for fostering the adoption of family-centred telehealth interventions in pediatric rehabilitation: A pan-Canadian implementation-effectiveness study.

Primary Inst./Inst. principal: Services et politiques de la santé

Other Related Inst./ Autres inst. connexes: Développement et santé des enfants et des adolescents

Competition /Concours: Subvention Projet
April/Avril 01, 2021

Number in competition/Nbre de demandes dans le concours: 2381

Peer Review Committee Recommendation, for your information and use/ Recommandation du comité d'examen par les pairs, pour fins d'information et d'utilisation:

**Committee/Comité:**	Recherche sur l'application des connaissances
**Number reviewed/ Demandes examinées:**	23
**Application rank within the committee/ Rang de la demande dans le comité:**	3
**Percent Rank within the committee / Rang en pourcentage au sein du comité:**	90.91%
**Rated / Cote:**	4.40
**Recommended Term/ Durée recommandée:**	4 years/ans 0 months/mois
**Recommended average annual operating amount/ Montant annuel moyen recommandé pour le fonctionnement:**	$245,000
**Recommended equipment amount/ Montant recommandé pour les appareils:**	$0

This document is for information only.
An application rated below 3.50 is ineligible for CIHR funding. For applications rated 3.50 and above, please note that it is the application's rank within the peer review committee that determines whether it is funded, rather than its absolute rating. The final funding decision will be communicated in the Notice of Decision.

Document à titre d'information seulement.
Une demande cotée en dessous de 3,5 n'est pas admissible au financement des IRSC. En ce qui a trait aux demandes cotées 3,50 ou plus, veuillez noter que l'on détermine l'attribution des fonds en fonction du classement obtenu au sein du comité d'examen par les pairs plutôt qu'en fonction du classement absolu. La décision finale relative au financement sera communiquée dans l'Avis de décision.

**Review Type / Type d'évaluation:**	Reviewer 1 / Évaluateur 1
**Name of Applicant / Nom du chercheur:**	Camden, Chantal
**Application No. / Numéro de demande:**	462626
**Agency / Agence:**	CIHR/IRSC
**Competition / Concours:**	Project Grant/Subvention Projet
**Committee / Comité:**	Knowledge Translation Research/Recherche sur l'application des connaissances
**Title / Titre:**	Training Intervention and Program of Support (TIPS) for fostering the adoption of family-centred telehealth interventions in pediatric rehabilitation: A pan-Canadian implementation-effectiveness study.

Adjudication Criteria/Critères de sélection

Initial Score/Cote Initiale: 4.0

Top/Bottom Selection/Groupe supérieur/inférieur

- (●) **Top/Groupe supérieur**
- ( ) **Bottom/Groupe inférieur**

**Review Type / Type d'évaluation:**	Reviewer 1 / Évaluateur 1
**Name of Applicant / Nom du chercheur:**	Camden, Chantal
**Application No. / Numéro de demande:**	462626
**Agency / Agence:**	CIHR/IRSC
**Competition / Concours:**	Project Grant/Subvention Projet
**Committee / Comité:**	Knowledge Translation Research/Recherche sur l'application des connaissances
**Title / Titre:**	Training Intervention and Program of Support (TIPS) for fostering the adoption of family-centred telehealth interventions in pediatric rehabilitation: A pan-Canadian implementation-effectiveness study.

Summary of Application/Résumé de la demande:

This proposal builds on and extends a substantial body of work on family-centred telehealth, notably a pilot project in Quebec of TIPS – Training Intervention and Program of Support - an intervention to help therapists adopt and adapt the "essential ingredients" of Family Centred Telehealth with families of children (0-12) with or at risk of disability. The intervention consists of 4 hours of self-paced modules for rehabilitation therapists, a 6-hour webinar about how to implement FCT practices, a year-long mentorship programme, and access to a virtual Community of Practice. The researchers propose a mixed methods implementation-effectiveness time series study design with progressive implementation in 20 sites across Canada. The study includes repeated self-administered questionnaires/measures to therapists about intention to adopt and adapt FCT, actual practice immediately, over time and after a year, documentation of meeting notes with the sites/teams, interviews with managers, interviews with family members regarding their satisfaction with the telehealth services, and of additional costs or savings related to increased use of FCT. The project is directly related to previous and current work but does not duplicate it. The project – over 4 years - will allow the Quebec pilot to be extended across Canada, over time.

**Review Type / Type d'évaluation:**	Reviewer 1 / Évaluateur 1
**Name of Applicant / Nom du chercheur:**	Camden, Chantal
**Application No. / Numéro de demande:**	462626
**Agency / Agence:**	CIHR/IRSC
**Competition / Concours:**	Project Grant/Subvention Projet
**Committee / Comité:**	Knowledge Translation Research/Recherche sur l'application des connaissances
**Title / Titre:**	Training Intervention and Program of Support (TIPS) for fostering the adoption of family-centred telehealth interventions in pediatric rehabilitation: A pan-Canadian implementation-effectiveness study.

Strengths and Weaknesses/Forces et faiblesses:

Concept

This is an exciting and very timely initiative given the confluence of increasing attention to patient/family-centred care, COVID-19 and its attendant shifts to tele-everything, and disability. Because of its explicit attention to how to identify and communicate “essential ingredients” of an intervention together with close attention to what needs to be adapted to specific contexts, and how, this work has the potential to influence not only family-centred telehealth for children with or at risk of disability, but to go beyond increasingly sterile debates about fidelity “versus” adaptation of complex interventions, and the attendant evaluation and approaches to scale-up of these. Within the substantive area and the field in Canada, it appears to reach a very significant proportion of key actors across the country. The design is very strong and the proposed instruments should enable findings to emerge that can be applied to other fields and contexts – that is to say, to inform and influence not only this field, but KTR much more broadly.

The relative weaknesses conceptually and methodologically are that 1) the proposal does not take full advantage of this opportunity to inform/transform the broader field, and 2) it is a very dense proposal which takes a lot of work. This is a weakness because it is not entirely obvious that the rich and complex lessons will be shared as accessibly as might be liked. I would have liked to see quite a bit more about how context is explored and addressed in relation to adaptation – including re cultural safety, gender, stigma, and economic/social and health realities and burdens. The instruments appear to focus mostly on how individual therapists, managers and family members experience and act on the intervention and only in relation to its “family centredness”; indeed, the only example of “contextual drivers” from the therapists’ perspectives is “technology availability”. It would have been helpful to include a broader socio-cultural perspective, including some explicit attention to even the basic descriptions and demographics of the 20 settings and to the (gendered) nature and potential precarity of work as well as care, or at least to be explicitly open to these elements emerging in the exploration of how “family centred” or how successful the intervention was. I would have also liked to see more about whether the FCT approach creates or lessens (gendered) burdens on therapists – will highly family-centred therapists feel or be considered by families to be duty-bound to go “above and beyond”? Do therapists have adequate emotional and other support (through the mentorship programme or elsewhere) for the additional emotional work that a truly person-centred and family-centred approach entails? However, in general I think this is an excellent, important and timely project. The hypotheses are clear, provocative, and again suggest a potentially transformative outcome/learning – including if the intervention “fails”, even spectacularly.

Feasibility - As the proposal notes, this is a complex project with a VERY large and complex team, which means that challenges in coordination and implementation are all but inevitable. The team is aware of this and has put in place necessary measures to ensure the tight coordination and communication which will be essential to its successful implementation. These are adequately budgeted. The team skills are appropriate, strong and complementary. There appears to be reasonable buy-in from the various proposed sites.

**Review Type / Type d'évaluation:**	Reviewer 1 / Évaluateur 1
**Name of Applicant / Nom du chercheur:**	Camden, Chantal
**Application No. / Numéro de demande:**	462626
**Agency / Agence:**	CIHR/IRSC
**Competition / Concours:**	Project Grant/Subvention Projet
**Committee / Comité:**	Knowledge Translation Research/Recherche sur l'application des connaissances
**Title / Titre:**	Training Intervention and Program of Support (TIPS) for fostering the adoption of family-centred telehealth interventions in pediatric rehabilitation: A pan-Canadian implementation-effectiveness study.

Budget Recommendation/Recommandation budgétaire:

The budget is substantial but appears appropriate.

**Review Type / Type d'évaluation:**	Reviewer 1 / Évaluateur 1
**Name of Applicant / Nom du chercheur:**	Camden, Chantal
**Application No. / Numéro de demande:**	462626
**Agency / Agence:**	CIHR/IRSC
**Competition / Concours:**	Project Grant/Subvention Projet
**Committee / Comité:**	Knowledge Translation Research/Recherche sur l'application des connaissances
**Title / Titre:**	Training Intervention and Program of Support (TIPS) for fostering the adoption of family-centred telehealth interventions in pediatric rehabilitation: A pan-Canadian implementation-effectiveness study.

Please indicate your appraisal of the integration of sex as a biological variable as a strength, weakness, or not applicable to the proposal./Prière de sélectionner une option pour donner votre évaluation de l'intégration du sexe comme variable biologique en tant que point fort ou point faible de la proposition, ou en tant qu'élément non applicable à la proposition.

- (●) **Strength/Point fort**
- ( ) **Weakness/Point faible**
- ( ) **Not applicable/Non applicable**

Please indicate your appraisal of the integration of gender as a socio-cultural determinant of health as a strength, weakness, or not applicable to the proposal./Prière de sélectionner une option pour donner votre évaluation de l'intégration du genre comme déterminant socioculturel de la santé en tant que point fort ou point faible de la proposition, ou en tant qu'élément non applicable à la proposition.

- (●) **Strength/Point fort**
- ( ) **Weakness/Point faible**
- ( ) **Not applicable/Non applicable**

Sex and/or Gender Considerations/Notions de sexe et/ou de genre:

Sex and gender are addressed thoughtfully and reasonably well, though of course sex and gender as well as other dimensions of identity, social location, vulnerability etc are likely to be relevant in much wider and deeper ways than have been explicitly articulated. As noted above: I would have liked to see quite a bit more about how context is explored and addressed in relation to adaptation of FCT – including re cultural safety, gender, stigma, and economic/social and health realities and burdens. The instruments appear to focus mostly on how individual therapists, managers and family members experience and act on the intervention and only in relation to its “family centredness”; indeed, the only example of “contextual drivers” from the therapists’ perspectives is “technology availability”. It would have been helpful to include a broader socio-cultural perspective, including some explicit attention to even the basic descriptions and demographics of the 20 settings and to the (gendered) nature and potential precarity of work as well as care, or at least to be explicitly open to these elements emerging in the exploration of how “family centred” or how successful the intervention was. I would have also liked to see more about whether the FCT approach creates or lessens (gendered) burdens on therapists – will highly family-centred therapists feel or be considered by families to be duty-bound to go “above and beyond”? Do therapists have adequate emotional and other support (through the mentorship programme or elsewhere) for the additional emotional work that a truly person-centred and family-centred approach entails?

**Review Type / Type d'évaluation:**	Reviewer 2 / Évaluateur 2
**Name of Applicant / Nom du chercheur:**	Camden, Chantal
**Application No. / Numéro de demande:**	462626
**Agency / Agence:**	CIHR/IRSC
**Competition / Concours:**	Project Grant/Subvention Projet
**Committee / Comité:**	Knowledge Translation Research/Recherche sur l'application des connaissances
**Title / Titre:**	Training Intervention and Program of Support (TIPS) for fostering the adoption of family-centred telehealth interventions in pediatric rehabilitation: A pan-Canadian implementation-effectiveness study.

Adjudication Criteria/Critères de sélection

Initial Score/Cote Initiale: 4.1

Top/Bottom Selection/Groupe supérieur/inférieur

- (●) **Top/Groupe supérieur**
- ( ) **Bottom/Groupe inférieur**

**Review Type / Type d'évaluation:**	Reviewer 2 / Évaluateur 2
**Name of Applicant / Nom du chercheur:**	Camden, Chantal
**Application No. / Numéro de demande:**	462626
**Agency / Agence:**	CIHR/IRSC
**Competition / Concours:**	Project Grant/Subvention Projet
**Committee / Comité:**	Knowledge Translation Research/Recherche sur l'application des connaissances
**Title / Titre:**	Training Intervention and Program of Support (TIPS) for fostering the adoption of family-centred telehealth interventions in pediatric rehabilitation: A pan-Canadian implementation-effectiveness study.

Summary of Application/Résumé de la demande:

This proposal aims to conduct a hybrid implementation-effectiveness study of a training and support intervention for family centred telehealthcare. The healthcare context is specific to pediatric rehabilitation but the 7 telehealth key ingredients could be adapted for other populations. The training and support intervention includes self-directed online education modules, interactive webinar, 12 month mentoring meetings and community of practice. A mixed-methods evaluation will be conducted involving clinician measures and insights, organizational service indicators, family service satisfaction, and costing analysis.

**Review Type / Type d'évaluation:**	Reviewer 2 / Évaluateur 2
**Name of Applicant / Nom du chercheur:**	Camden, Chantal
**Application No. / Numéro de demande:**	462626
**Agency / Agence:**	CIHR/IRSC
**Competition / Concours:**	Project Grant/Subvention Projet
**Committee / Comité:**	Knowledge Translation Research/Recherche sur l'application des connaissances
**Title / Titre:**	Training Intervention and Program of Support (TIPS) for fostering the adoption of family-centred telehealth interventions in pediatric rehabilitation: A pan-Canadian implementation-effectiveness study.

Strengths and Weaknesses/Forces et faiblesses:

This is an exciting proposal because the intervention is potentially highly generalizable across healthcare, and thus relevant to study how to optimally implement it. The family-centred telehealthcare model at the core of the study is based on strong foundational evidence and the training and program support intervention to adopt family-centred telehealthcare has been rigorously developed and evaluated. An established integrated KT approach is central to the proposal. A comprehensive evaluation plan is proposed, including a cost analysis.

I do think that the research design could be strengthened. Although the applicants have justified reasons for a pre-post design, given the number of sites, a stepped wedge would seem very feasible and randomizing start dates would offer an additional control analysis. I also thought the specific implementation research questions could be strengthened and there is lots of opportunity for comparative analysis, and this was a missed opportunity. For example, why is the same TIPS intervention proposed for sites with high and low intention to adopt? Would we not hypothesize that tailored strategies may increase adoption and these could therefore be compared? I’m sure there are many other characteristics and issues that could be explored within this grant. I encourage the applicants to more clearly define these.

With regard to feasibility, have all the KUs been recruited to the Local Leadership Team at each site? This seems important, and I did not get this impression from all of the support letters.

It was not clear how 20 sites were chosen as the sample size number? Was it intended to be representative? Or based on caseloads is this how many sites it will take to reach sample size targets?

**Review Type / Type d'évaluation:**	Reviewer 2 / Évaluateur 2
**Name of Applicant / Nom du chercheur:**	Camden, Chantal
**Application No. / Numéro de demande:**	462626
**Agency / Agence:**	CIHR/IRSC
**Competition / Concours:**	Project Grant/Subvention Projet
**Committee / Comité:**	Knowledge Translation Research/Recherche sur l'application des connaissances
**Title / Titre:**	Training Intervention and Program of Support (TIPS) for fostering the adoption of family-centred telehealth interventions in pediatric rehabilitation: A pan-Canadian implementation-effectiveness study.

Budget Recommendation/Recommandation budgétaire:

Appropriate

**Review Type / Type d'évaluation:**	Reviewer 2 / Évaluateur 2
**Name of Applicant / Nom du chercheur:**	Camden, Chantal
**Application No. / Numéro de demande:**	462626
**Agency / Agence:**	CIHR/IRSC
**Competition / Concours:**	Project Grant/Subvention Projet
**Committee / Comité:**	Knowledge Translation Research/Recherche sur l'application des connaissances
**Title / Titre:**	Training Intervention and Program of Support (TIPS) for fostering the adoption of family-centred telehealth interventions in pediatric rehabilitation: A pan-Canadian implementation-effectiveness study.

Please indicate your appraisal of the integration of sex as a biological variable as a strength, weakness, or not applicable to the proposal./Prière de sélectionner une option pour donner votre évaluation de l'intégration du sexe comme variable biologique en tant que point fort ou point faible de la proposition, ou en tant qu'élément non applicable à la proposition.

- ○ **Strength/Point fort**
- ◉ **Weakness/Point faible**
- ○ **Not applicable/Non applicable**

Please indicate your appraisal of the integration of gender as a socio-cultural determinant of health as a strength, weakness, or not applicable to the proposal./Prière de sélectionner une option pour donner votre évaluation de l'intégration du genre comme déterminant socioculturel de la santé en tant que point fort ou point faible de la proposition, ou en tant qu'élément non applicable à la proposition.

- ◉ **Strength/Point fort**
- ○ **Weakness/Point faible**
- ○ **Not applicable/Non applicable**

**Review Type / Type d'évaluation:**	Reviewer 2 / Évaluateur 2
**Name of Applicant / Nom du chercheur:**	Camden, Chantal
**Application No. / Numéro de demande:**	462626
**Agency / Agence:**	CIHR/IRSC
**Competition / Concours:**	Project Grant/Subvention Projet
**Committee / Comité:**	Knowledge Translation Research/Recherche sur l'application des connaissances
**Title / Titre:**	Training Intervention and Program of Support (TIPS) for fostering the adoption of family-centred telehealth interventions in pediatric rehabilitation: A pan-Canadian implementation-effectiveness study.

Sex and/or Gender Considerations/Notions de sexe et/ou de genre:

Gender considerations are appropriate. I am unclear how the biological variable of sex relates to this proposal.

**Review Type / Type d'évaluation:**	Reviewer 3 / Évaluateur 3
**Name of Applicant / Nom du chercheur:**	Camden, Chantal
**Application No. / Numéro de demande:**	462626
**Agency / Agence:**	CIHR/IRSC
**Competition / Concours:**	Project Grant/Subvention Projet
**Committee / Comité:**	Knowledge Translation Research/Recherche sur l'application des connaissances
**Title / Titre:**	Training Intervention and Program of Support (TIPS) for fostering the adoption of family-centred telehealth interventions in pediatric rehabilitation: A pan-Canadian implementation-effectiveness study.

Adjudication Criteria/Critères de sélection

Initial Score/Cote Initiale: 4.3

Top/Bottom Selection/Groupe supérieur/inférieur

- ◉ **Top/Groupe supérieur**
- ◯ **Bottom/Groupe inférieur**

**Review Type / Type d'évaluation:**	Reviewer 3 / Évaluateur 3
**Name of Applicant / Nom du chercheur:**	Camden, Chantal
**Application No. / Numéro de demande:**	462626
**Agency / Agence:**	CIHR/IRSC
**Competition / Concours:**	Project Grant/Subvention Projet
**Committee / Comité:**	Knowledge Translation Research/Recherche sur l'application des connaissances
**Title / Titre:**	Training Intervention and Program of Support (TIPS) for fostering the adoption of family-centred telehealth interventions in pediatric rehabilitation: A pan-Canadian implementation-effectiveness study.

Summary of Application/Résumé de la demande:

The goal of this project is to evaluate the implementation of a training program to support family centered telehealth rehab therapist services for families/children. *how best to support pediatric therapists so that telehealth becomes a sustainable option used by therapists*

Rationale: 10% of Canadian parents are caring for a child with a disability. Rehab needs were not being met even pre-COVID. There have been knowledge to practice gaps in implementation of telehealth rehab services. Telehealth was adopted, but without adequate support. Training and supports for effective telehealth will help sustain these services as part of family-centered model. Opportunity to learn from this and address need.

The primary objective in this study is to evaluate the implementation (adoption, fidelity of practices). Secondary objectives are to identify contextual variations required, identify factors influencing adoption/fidelity, evaluate effectiveness of the program.

METHODS: This is a mixed methods implementation-effectiveness study with 20 recruited sites (various pop, size, services across 8 provinces); manager (1), therapists and patients (parents) at each site: 600 therapists, 12000 eligible parents (to expected sample from 300 therapists who have interest in using FCT at least 1/wk, 2000 parents per assessment point).

ITS design outcomes are therapist self-reported: 1. Intention to adopt, 2. Adoption, 3. Fidelity, 4. Engagement practices. Four follow-up periods. Primary question is if TIPS can be co-adapted in different contexts. Secondary outcomes are proposed to evaluate important outcomes of effectiveness (limited to wait times, perceived quality of services, changes in service delivery) and costs (limited description of measurement: by journal and collected from families).

Qualitative data collection at team meetings and post-TIPS interviews with therapists, managers, families (all managers, selected sample of therapists, families) guided by CFIR domains. Planned integration of qualitative with quantitative.

**Review Type / Type d'évaluation:**	Reviewer 3 / Évaluateur 3
**Name of Applicant / Nom du chercheur:**	Camden, Chantal
**Application No. / Numéro de demande:**	462626
**Agency / Agence:**	CIHR/IRSC
**Competition / Concours:**	Project Grant/Subvention Projet
**Committee / Comité:**	Knowledge Translation Research/Recherche sur l'application des connaissances
**Title / Titre:**	Training Intervention and Program of Support (TIPS) for fostering the adoption of family-centred telehealth interventions in pediatric rehabilitation: A pan-Canadian implementation-effectiveness study.

Strengths and Weaknesses/Forces et faiblesses:

STRENGTHS:

-Extension of previous work of the team to develop the TIPS telehealth support program and opportunity to capitalize on the COVID related momentum for telehealth services (4% using telehealth in 2019; 70% in 2020). The TIPS program was developed based on systematic review of RCTs conducted by the team. The team has pilot tested the training program in Quebec and now propose to scale up across Canada. Program includes learning modules, webinar, 1-year mentoring meetings and community of practice. 20 rehab sites are already recruited for current proposed study.

-TIPS program and supports being offered in French and English language of choice.

-For therapist implementation – good measurement of intention, actual implementation; nice assessment of fidelity. Very complete and robust.

-Nice comprehensive and theoretically guided assessment of local implementation and adaptation guided by CIFR with questionnaire (open and closed ended Q). Qualitative assessment of local team/mentoring, and national meeting recordings and community of practice threads (unclear value of this and unclear methods toward goal to identify factors related to intention to adopt… are there questions/discussion planned in these meeting?)

-Integrated KT plan with parent-partners at steering committee and local teams. Proposes online forums (annually) for broader public engagement (how? And with what purpose?)

-Glad to see that the team raises concerns about inequity with the intervention (but don't measure this potential adverse effect). Mentions that culture, sex, gender will be considered as factors that influence adaptation and implementation of TIPS but unclear how or if quantitative analyses are powered to do this.

-Good KT - sharing through website and accessible by partners sites and newsletters and webinars at local sites. Focus on this would be more helpful than publications.

-I appreciate and compliment the team on the extensive background preparation and documentation for this project with letters of support and detailed description of tools in appendix document. Some tools have been developed for other projects – assume these will need to be modified (how will fidelity tool be adapted – no mention of video recording)?

-NPI is a midcareer researcher with exceptional track record. Team includes a large complementary team with mix of early career, mid, and senior researchers (KT, rehab, telehealth expertise); parent/patient partners; planned involvement of trainees (clinician scientists); KUs (11 partner organizations) are appropriate and committed; represent children's healthcare network leaders. Many team members have collaborated before on related projects. NPI has a successful track record of funding, collaborations and productivity. Several recent projects directly inform this current proposed study (national survey; fidelity checklist validation study). The team includes long-term research staff with relevant experience. Expertise and support includes data collection with REDCap, SPOR support unit, statistics, involvement of local clinical champions, thoughtful governance/engagement structure. **Positive contributions to interprovincial linkages and national support network for ped rehab.

WEAKNESSES:

-Effectiveness trial for the TIPS intervention being proposed (developed based on literature review) is still ongoing (stated

as available in late 2021; reports “Preliminary results are promising”). Evidence of effectiveness? The proposal argues that early implementation is justified by current context momentum, evidence for telehealth/training in general and feasibility; promising early results for TIPS.

-Challenge of ITS study design with current changing pandemic restrictions lifting. Will be very difficult to assess – limitation.

-Good rationale for research area. Feel like the proposed study focuses on earlier stage of implementation process than argued by the background/rationale rather than effectiveness of implementation (includes this only as secondary objective and not with best measures). If hesitation about if the TIPS program can be adapted then is it too early for effectiveness study? Do the preliminary work to see if adaptation is possible with a subsequent study focused on effectiveness of implementation.

-Limitations: Process outcomes measures only in ITS with multiple pre-post timepoints (therapist questionnaire – intention, fidelity). Other outcomes are pre-post only with subset. Objectives 4 & 5 (effectiveness, cost) are administered to only a subsample of the parent population (selection bias, measurement bias are concerns). It will be very difficult to draw conclusions from the effectiveness questions due to potential bias in sampling, measurement.

-PRIME-SP questionnaire refers to a specific patient-provider interaction – how will these interactions be selected for completion? All patients for recruited HCPs? Only recruited patients of recruited HCPs? Is there potential selection bias with these outcomes as well as for effectiveness outcomes?

-Fidelity questionnaire in appendix seems to be based on review of video of a patient interaction. It is unclear how this will be used in the current study.

-Description of analysis of qualitative data is limited - simply describes plan to use a deductive-inductive approach guided by CFIR domains. Qualitative integrated with quantitative (no detail).

-Limitations/mitigation strategies section is incomplete – discusses potential ceiling effect for intention to adopt. This is dismissed as earlier report suggested therapists feel low self-perceived efficacy. This has maybe changed since early in the pandemic. Other potential limitations? Mixed methods gets at primary implementation process outcomes, but not really effectiveness, which is assessed in a limited way.

-Is there patient-engagement?

-Study design has limitations for assessment of impact. But still useful for identifying contextual issues for implementation (and low risk).

**Review Type / Type d'évaluation:**	Reviewer 3 / Évaluateur 3
**Name of Applicant / Nom du chercheur:**	Camden, Chantal
**Application No. / Numéro de demande:**	462626
**Agency / Agence:**	CIHR/IRSC
**Competition / Concours:**	Project Grant/Subvention Projet
**Committee / Comité:**	Knowledge Translation Research/Recherche sur l'application des connaissances
**Title / Titre:**	Training Intervention and Program of Support (TIPS) for fostering the adoption of family-centred telehealth interventions in pediatric rehabilitation: A pan-Canadian implementation-effectiveness study.

Budget Recommendation/Recommandation budgétaire:

Appropriate and thoughtful budget for research staff and trainees.

6 open access publications seems like unhelpful splitting of study results. Protocol could be open access without APC, 2-3 publications.

Please indicate your appraisal of the integration of sex as a biological variable as a strength, weakness, or not applicable to the proposal./Prière de sélectionner une option pour donner votre évaluation de l'intégration du sexe comme variable biologique en tant que point fort ou point faible de la proposition, ou en tant qu'élément non applicable à la proposition.

- ○ **Strength/Point fort**
- ◉ **Weakness/Point faible**
- ○ **Not applicable/Non applicable**

Please indicate your appraisal of the integration of gender as a socio-cultural determinant of health as a strength, weakness, or not applicable to the proposal./Prière de sélectionner une option pour donner votre évaluation de l'intégration du genre comme déterminant socioculturel de la santé en tant que point fort ou point faible de la proposition, ou en tant qu'élément non applicable à la proposition.

- ○ **Strength/Point fort**
- ◉ **Weakness/Point faible**
- ○ **Not applicable/Non applicable**

**Review Type / Type d'évaluation:**	Reviewer 3 / Évaluateur 3
**Name of Applicant / Nom du chercheur:**	Camden, Chantal
**Application No. / Numéro de demande:**	462626
**Agency / Agence:**	CIHR/IRSC
**Competition / Concours:**	Project Grant/Subvention Projet
**Committee / Comité:**	Knowledge Translation Research/Recherche sur l'application des connaissances
**Title / Titre:**	Training Intervention and Program of Support (TIPS) for fostering the adoption of family-centred telehealth interventions in pediatric rehabilitation: A pan-Canadian implementation-effectiveness study.

Sex and/or Gender Considerations/Notions de sexe et/ou de genre:

The application acknowledges the importance of both sex and gender in decision making, stakeholder engagement, communication, preferences and uptake of behavioural interventions and knowledge translation (KT) strategies. In the brief section, the application describes that they will collect and describe both and consider the impact on training needs. They acknowledge limitations of their sample (mostly female caregivers and providers), but express desire for representation and tailoring of resulting KT messages/tools. Unclear how these will be achieved.

SGBA in the application methods is limited – gender is mentioned as one covariate for secondary analysis to explore heterogeneity for primary question (no mention in sample size). Appears to be measured in the ADOPT-VR survey (described as “Gender: M, F”; note language misuse: male/female refers to biological sex; man/woman/non-binary refers to gender).

Review Type/Type d'évaluation: SO Notes /Notes de l'agent scientifique
Name of Applicant/Nom du chercheur: Camden, Chantal
Application No./Numéro de demande: 462626
Agency/Agence: CIHR/IRSC
Competition/Concours: 2021-04-01 Project Grant/Subvention Projet
Committee/Comité: Knowledge Translation Research/Recherche sur l'application des connaissances
Title/Titre: Training Intervention and Program of Support (TIPS) for fostering the adoption of family-centred telehealth interventions in pediatric rehabilitation: A pan-Canadian implementation-effectiveness study.

Assessment/Évaluation:

Strengths (including SGBA considerations):

This is a timely and important project, particularly given emphasis on patient/family-centred care and move to virtual care during COVID-19 pandemic. Well-presented project with the potential to influence work in this field but outside in other content areas also.

Reviewers were excited to see this proposal. Now is the time to take advantage of and learn more about use of telehealth.

Good to see a focus on fidelity vs adaptation and what the “essential ingredients” of the intervention are. This is a strength.

The overall project approach was strong. The proposed instruments are appropriate and should be able to help influence KTE field more broadly.

The team has expertise required to complete project. The PI is very productive, and this project builds directly on work she has done.

This project has huge potential to be generalizable to other contexts and settings (the lessons learned can be applied to many situations/settings).

This is a very complex project – however, the team is aware of this and has put strategies in place to enable good coordination, etc.

There appears to have good buy-in from proposed sites, with authentic and appropriate letters of support /collaborations, and thus the team should be well positioned to carry out this project.

Sex and gender are addressed reasonably well.

Review Type/Type d'évaluation: SO Notes /Notes de l'agent scientifique
Name of Applicant/Nom du chercheur: Camden, Chantal
Application No./Numéro de demande: 462626
Agency/Agence: CIHR/IRSC
Competition/Concours: 2021-04-01 Project Grant/Subvention Projet
Committee/Comité: Knowledge Translation Research/Recherche sur l'application des connaissances
Title/Titre: Training Intervention and Program of Support (TIPS) for fostering the adoption of family-centred telehealth interventions in pediatric rehabilitation: A pan-Canadian implementation-effectiveness study.

Assessment/Évaluation:

Weaknesses (including SGBA considerations):

The proposal does not take the full opportunity to understand specific implementation research questions, such as fidelity vs adaptation and how those relate to context. There is so much that could be unpacked here in terms of learnings for implementation science.

It was a very dense proposal – the reviewers note that future KT should work on being less dense to ensure the findings are broadly communicated and understandable.

There were a number of limitations around study design:

· With 20 sites, moving to a stepped wedge would be possible and would offer a stronger design (and possibly make this a more feasible design since everything doesn't have to happen at once)

· A pre-post design will be used for assessment of the cost and effectiveness outcomes – though these are secondary outcomes, it is a weaker design than the ITS for the primary outcomes. Also these secondary outcomes will only be assessed for a subset of the parent population, so this may introduce selection bias.

· There was no justification for number of sites and why these particular sites were selected. Will these sites provide diversity in terms of patient population or geography? Who might the team be missing?

Reviewers would like to see more about how context will be explored in this study as it relates to adaptation – particularly regarding gender, cultural safety, stigma, etc. It would be helpful to include a broader socio-cultural perspective in the project.

Reviewers also noted wanting to see more about the burden, including the gendered burden, of the intervention on therapists themselves. Also, do therapists have adequate emotional support for the additional work they will be doing as part of this intervention?

Team seems to be limited in terms of expertise around costing analyses, though the team is lauded for identifying a

Review Type/Type d'évaluation: SO Notes /Notes de l'agent scientifique
Name of Applicant/Nom du chercheur: Camden, Chantal
Application No./Numéro de demande: 462626
Agency/Agence: CIHR/IRSC
Competition/Concours: 2021-04-01 Project Grant/Subvention Projet
Committee/Comité: Knowledge Translation Research/Recherche sur l'application des connaissances
Title/Titre: Training Intervention and Program of Support (TIPS) for fostering the adoption of family-centred telehealth interventions in pediatric rehabilitation: A pan-Canadian implementation-effectiveness study.

Assessment/Évaluation:

need to examine costs.

This project would provide excellent training opportunities and it would be great to see more trainees involved.

Budget:

No concerns.

****

Note: The final rating of the application, provided in the Notice of Recommendation (NOR) and Notice of Decision (NOD), is the averaged rating of the peer review committee members following the discussion of the application during the committee meeting, and therefore may differ from the ratings provided by the assigned reviewers in their respective reviews.

Remarque : La cote définitive de la demande, qui apparaît dans l'avis de recommandation et l'avis de décision, représente la moyenne des cotes accordées par les membres du comité d'évaluation par les pairs après avoir débattu de la demande à la réunion du comité. Elle peut donc différer de celle donnée par les évaluateurs dans leur évaluation respective.

````````````````````````````````````````````````````````````````````````````````````````````````````````````````````````
````````````````````````````````````````````````````````````````````````````````````````````````````````````````````````
